# Age influence on repetition suppression revealed in the hippocampus with MEG

**DOI:** 10.1002/alz70856_102845

**Published:** 2025-12-25

**Authors:** Melisa Gumus, Luis Garcia Dominguez, Wen Jia Zhao, Richard Wennberg, Michael L. Mack, Ljubica Zotovic, Krista L Lanctôt, Sandra E. Black, Mary Pat McAndrews

**Affiliations:** ^1^ University of Toronto, Toronto, ON, Canada; ^2^ Toronto Western Hospital, University Health Network, Toronto, ON, Canada; ^3^ Sunnybrook Research Institute, Toronto, ON, Canada

## Abstract

**Background:**

One candidate biomarker for Alzheimer's Disease (AD) is the hyperexcitability of the hippocampus in presymptomatic stages or Mild Cognitive Impairment that transforms into hypoactivation with disease progression. A potential tool to measure this neural shift is repetition suppression, defined as the neural tuning over repeated information during memory formation. Yet, to establish such a neural biomarker, it is important to characterize the aging effects on repetition suppression in the hippocampus. By leveraging high temporal and spatial properties of magnetoencephalography (MEG), we examined distinct neural activation profiles in the hippocampus associated with repetition suppression in healthy young versus older adults, aiming to identify a promising avenue for a neural biomarker for AD.

**Methods:**

We collected MEG recordings from 53 participants, 27 healthy young (F=20, Age=18‐34) and 26 older adults (F=17, Age=60+), while they viewed series of scenes, where some images repeated after variable (2 to 8 item) lags. We aligned participant‐specific MEG data with their structural images and localized hippocampal signals through beamformer analyses. Repetition suppression was computed as the oscillatory difference between the novel and the repeated presentation of the images. In each group, we characterized the clusters of neural synchronies with time‐frequency analyses. We compared the probability of significant synchrony differences between the two groups against a null distribution in a permutation test.

**Results:**

Healthy older adults exhibited significant clusters of hippocampal alpha (8‐12Hz) and beta (13‐30Hz) desynchronizations for the repeated images in comparison to the novel (*p* <0.001), indicating more neural activity. The desynchronization of the hippocampus for the repeated images was not as pronounced in healthy young adults. In fact, permutation testing revealed that the extent of beta desynchronization for the repeated presentation was significantly greater in the hippocampus of healthy older adults than young (*p* <0.05).

**Conclusion:**

Neural tuning of the hippocampus during memory formation, namely repetition suppression, changes with age; expressed as greater neural activation in healthy older adults. Characterizing the aging effects on repetition suppression may offer a promising avenue for examination of hippocampal hyperexcitability as a potential biomarker for AD.

